# Anteromedial fractures of the ulnar coronoid process: correlation between surgical outcomes and radiographic findings

**DOI:** 10.1186/s12891-018-2162-z

**Published:** 2018-07-23

**Authors:** Alvin Chao-Yu Chen, Chun-Jui Weng, Ying-Chao Chou, Chun-Ying Cheng

**Affiliations:** Department of Orthopaedic Surgery, Bone and Joint Research Center, Chang Gung Memorial Hospital-Linkou & University College of Medicine, 5th, Fu-Shin St., Kweishan District, Taoyuan, 333 Taiwan, Republic of China

**Keywords:** Elbow, Instability, Coronoid fracture, Anteromedial facet, Lateral collateral ligament, Varus instability

## Abstract

**Background:**

This study aimed to report the radiographic findings and surgical outcomes of anteromedial facet (AMF) fracture of the ulnar coronoid process and to suggest an optimal approach.

**Methods:**

In this retrospective study, 20 consecutive patients with unilateral AMF fracture of coronoid process were surgically treated and divided into two groups without (group A) and with (group B) additional proximal ulnar fractures in equal case number. Time from injury to surgery averaged 4.38 ± 2.56 weeks. Mayo Elbow Performance Score (MEPS) and Shortened Disability of the Arm and Shoulder and Hand (quickDASH) score were used for functional evaluation. Cohen kappa coefficient (kappa) analysis was used to determine interobserver reliability on a radiographic reading.

**Results:**

All cases had a mean follow-up of 2.3 years. MEPS at 2 years averaged 87.75 ± 12.51; quickDASH, 7.05 ± 6.19. A significantly higher MEPS was found in subtype 3 than in subtype 2 (*p* = 0.036) and in group B than in group A (*p* = 0.020). Significantly lower quickDASH cores were found in group B than in group A (*p* = 0.011). Kappa analysis showed moderate agreement in the O’Driscoll classification (kappa = 0.56) and substantial agreement in categorization of the additional proximal ulnar fractures (kappa = 0.76).

**Conclusions:**

Additional proximal ulnar lesions were considered an integral part of varus posteromedial rotatory instability and required further categorization in the management of AMF fractures. Significantly better functional outcomes were achieved when those lesions were fully addressed.

## Background

Traditionally, the severity of coronoid fractures and their correlation with elbow stability were classified by fracture fragment size [1]. Previous studies have shown that the coronoid process consists of an anterior projection and an anteromedial facet (AMF). While the anterior projection was an anterior buttress of the elbow joint with > 25% involvement leading to gross instability [2–4], the AMF served as medial extension of the proximal ulna and was prone to fracture in resisting varus rotatory force [5, 6]. A more extensive classification system was proposed based on both fracture size and location to help analyze trauma mechanisms and predict associated injuries. However, coronoid fractures of the AMF, unlike those in terrible triad injuries, commonly show a diverse presentation [7]. Anatomically, fractures of the AMF may consist of simple avulsion of the sublime tubercle, a medial cortex split, and coronoid tip fracture with a tiny or large anterior fragment. The use of a strategic surgical approach is critical. The purpose of this study is to report the surgical outcomes of a consecutive case series and suggest an optimal surgical approach through a retrospective analysis of the surgical and radiographic findings of AMF fracture dislocation.

## Methods

Between 2007 and 2014, 20 patients with a displaced AMF fracture of the ulnar coronoid process and elbow subluxation were diagnosed with VPMRI by one orthopedic surgeon and one radiologist and referred for definite management. Chang Gung Institutional Review Board approval (IRB 201701326B0) was obtained for a retrospective review of all 20 patients’ records and radiographs. The report of patients’ data was in compliance with the Helsinki Declaration. Written consent to participate in the study was obtained from all the patients. Among them, 17 were primary injuries, while three were revision cases that had failed previous open reduction and internal fixation surgery. All patients underwent surgical fixation by the same surgeon, including five women and 15 men with an average age of 43.65 ± 12.17 years (range, 26–67 years). Twelve were in the left elbow; the other eight were in the right elbow. Three patients had concomitant ulnar neuropathy. The mean time interval from injury to surgery was 4.38 ± 2.56 weeks (range, 0–10). Based on radiographic findings, all 20 patients were divided into two groups. Patients without additional proximal ulnar lesions was in group A and served as a control; patients with additional proximal ulnar lesions, group B.

### Radiographic assessment

**Fig. 1:**
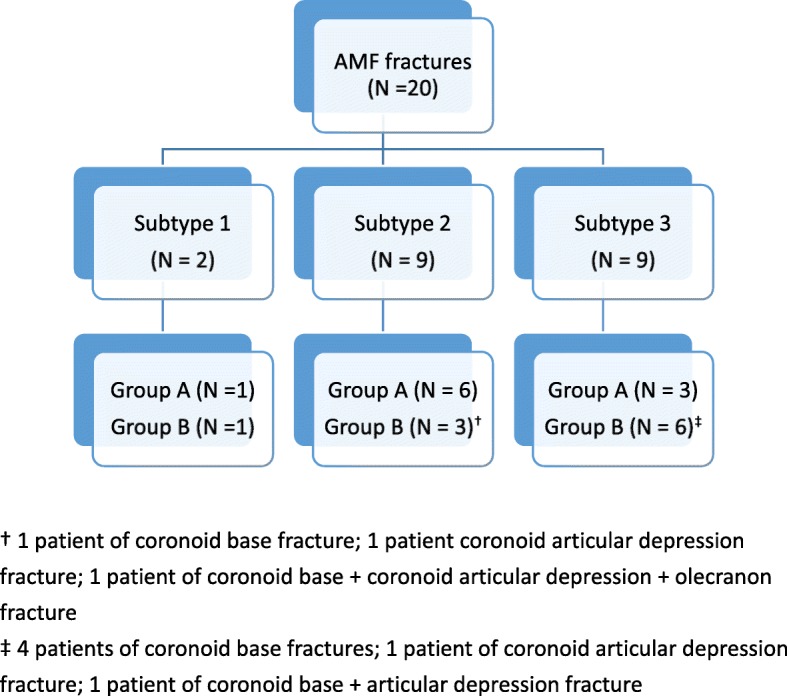
Patient allocation according to fracture patterns

**Fig. 2:**
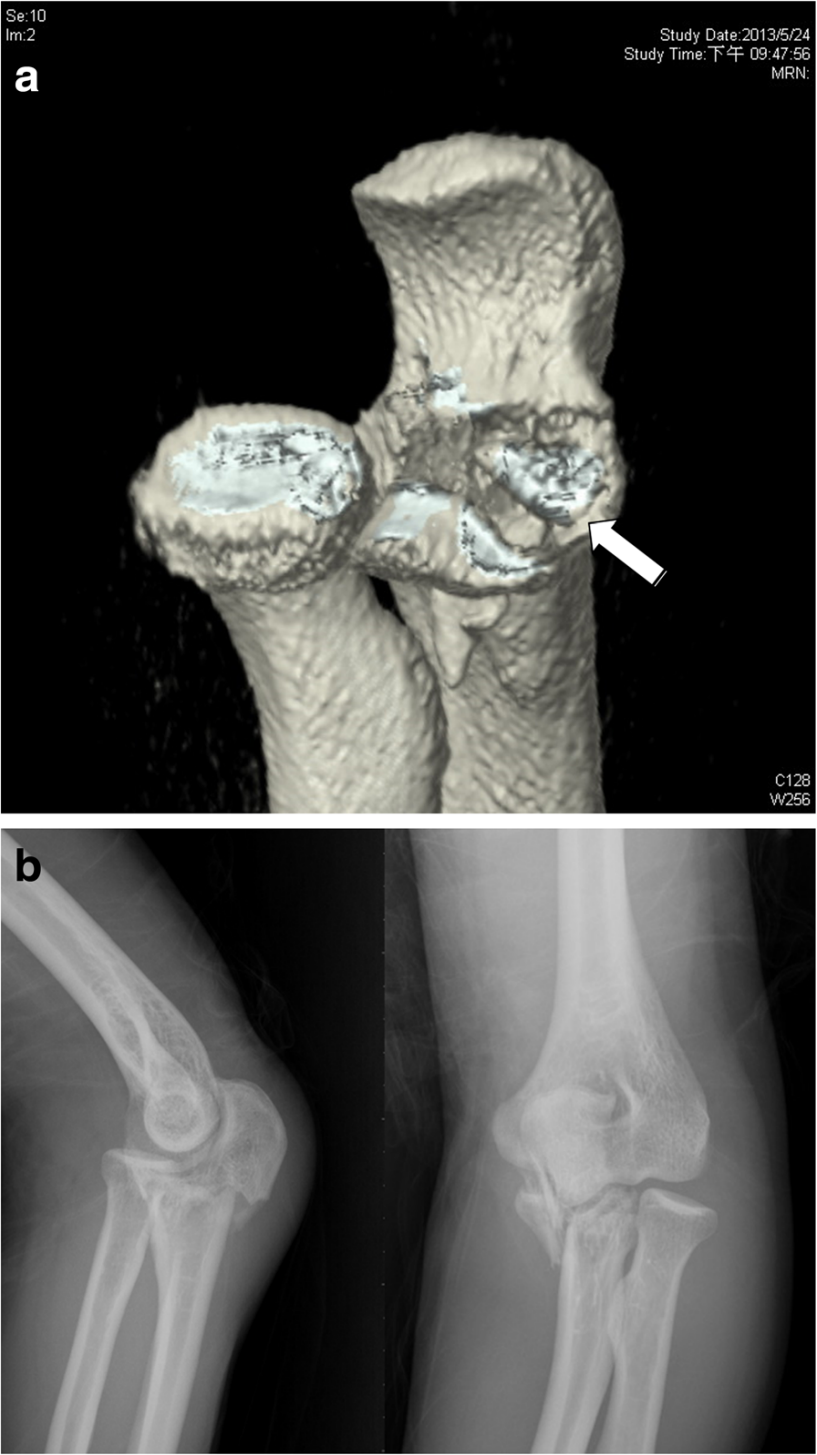
Two cases of anteromedial facet fractures. **a** Three-dimensional computed tomography scans showing an articular depression fracture (white arrow) close to the base of the coronoid process. **b** Plain radiographs showing an olecranon fracture with a displaced anteromedial facet fracture of the coronoid process

Preoperative plain X-ray and computed tomography (CT) images were retrospectively reviewed by two of the authors (one orthopedic trauma surgeon [WCJ] and one elbow surgeon [ACC]). Based on the O’Driscoll classification for AMF fracture, two were subtype 1, nine were subtype 2, and nine were subtype 3. Furthermore, additional fractures of the proximal ulna beyond the definition of the O’Driscoll classification were found in 10 cases (Fig. 1). A coronoid base fracture was found in one case of subtype 2 and three cases of subtype 3. An articular depression between the AMF and the coronoid base (Fig. 2a) was found in one case of subtype 2 and three cases of subtype 3. An olecranon fracture was found in three cases, one of each subtype (Fig. 2b). Accordingly, we divided the 20 cases into two radiographic groups: group A, 10 cases without additional fractures; and group B, 10 cases with additional fractures. The patients’ demographic data are listed in Table 1.

**Table 1:** Demographic data of patients

Characteristics	Group A	Group B
Mean age (years)	47	40.3
Gender		
Women	3	2
Men	7	8
O’Driscoll’s classification type 2 fracture		
Subtype 1	1	1
Subtype 2	7	3
Subtype 3	2	6
Time to surgery (weeks)	2.2	1.3

### Surgical strategy and technique

**Fig. 3:**
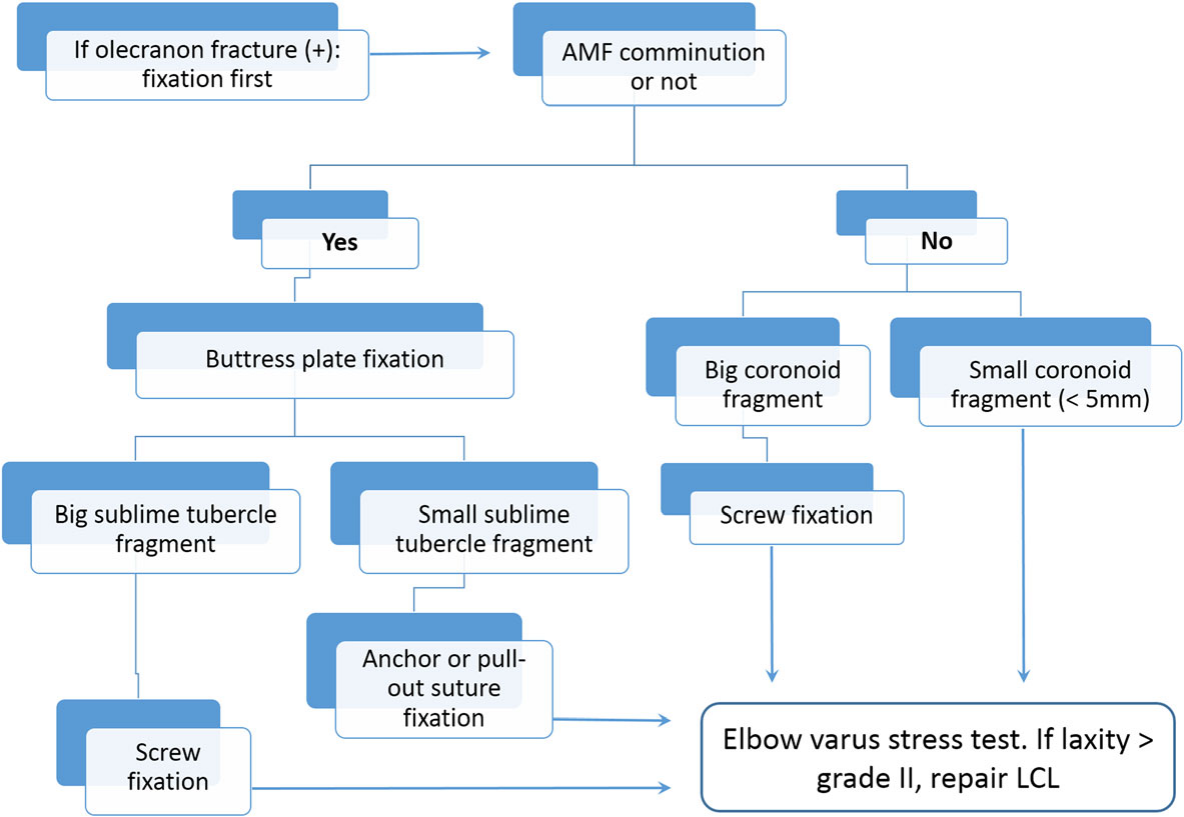
Treatment algorithm. AMF, anteromedial facet; LCL, lateral collateral ligament

**Fig. 4:**
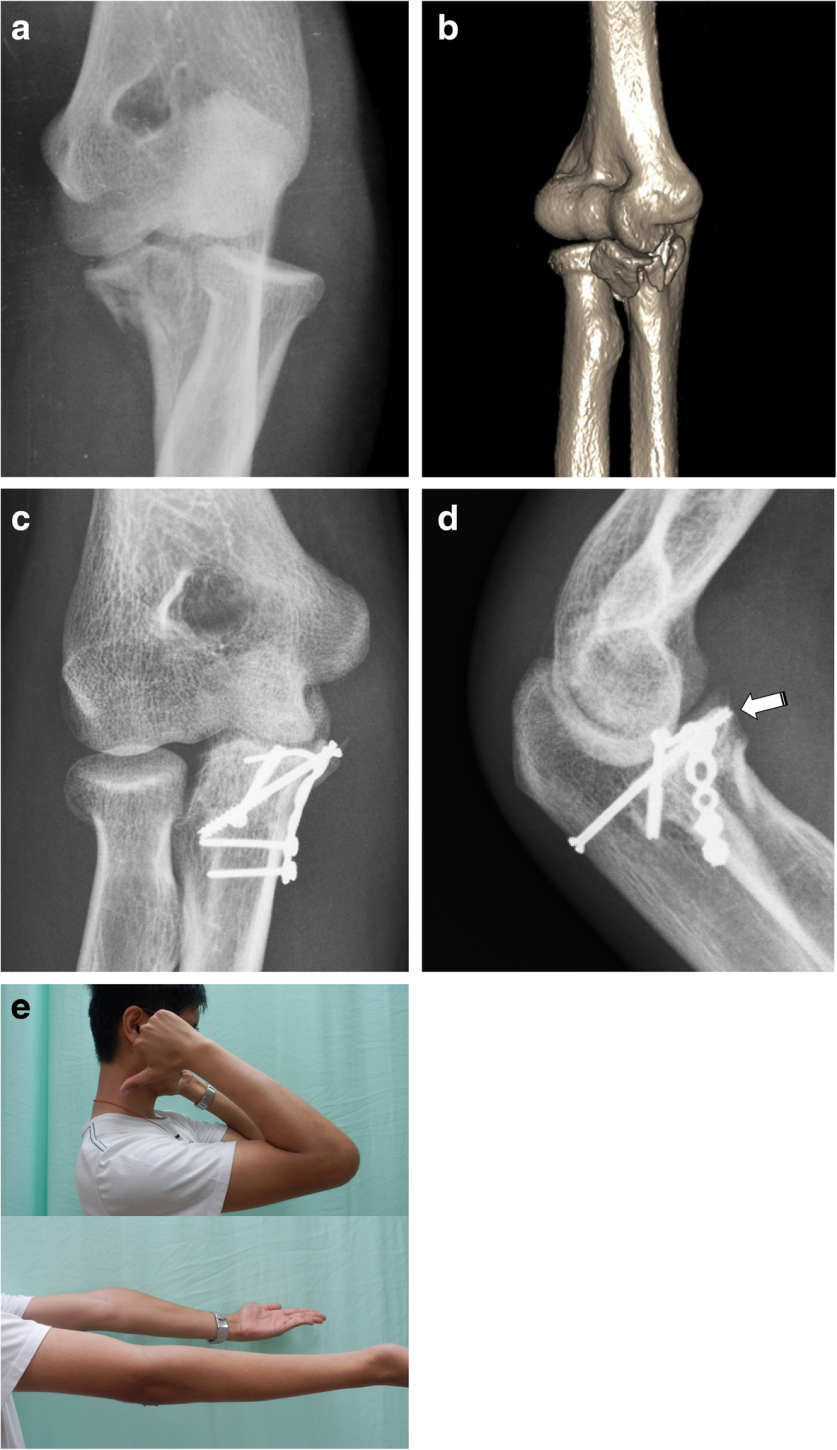
Anteromedial facet fracture with varus instability. **a** Plain radiograph. **b** Three-dimensional computed tomography image showing a large coronoid fragment. **c** Postoperative anteroposterior radiograph. **d** Postoperative lateral radiograph; the white arrow shows retrograde screw fixation of a coronoid base fracture. **e** Patient photo at 10 weeks after surgery showing good range of motion

Two kinds of surgical approaches for treating AMF of the coronoid process were used according to the preoperative radiographic analysis. For the three cases of olecranon fracture, a posterior global approach was adopted; for the other 17 cases, a medial over-the-top approach was used. A lateral incision was added whenever exposure of the lateral collateral ligament (LCL) was necessary. The surgical strategy was based on the O’Driscoll classification as well as the presentation of additional fractures (Fig. 3). To treat olecranon fracture, dorsal plating was applied for length and alignment restoration in two cases and a revision Kirschner wire with tension band fixation was performed in one case. A hybrid of internal fixation was used for coronoid fracture depending on the fracture patterns (Fig. 4). For large reducible fragments of the anterior coronoid without comminution, retrograde pinning followed by cannulated screw fixation from the dorsal proximal ulna was performed. Eighteen of our 20 cases (90%) showed an anteromedial rim split fracture accompanied by articular depression in four cases (20%) and was buttressed using a pre-contoured mini-plate (Synthes, Switzerland) or a Coronoid Plate (Acumed, OR, USA). The sublime tubercle avulsion fracture in nine cases (45%) was reduced and fixed with an interfragmental screw or suture anchor for securing the anterior band of the medial collateral ligament. After the fracture fixation, lateral elbow stability was checked under a mini-c-arm image intensifier. Three cases showed greater than grade II laxity and were treated with LCL repair using suture anchor fixation. In three cases (15%) of concomitant ulnar neuropathy with injury, surgical decompression and anterior transposition of the ulnar nerve was performed.

Postoperatively, the elbow was immobilized in a long arm splint at 90° of flexion and neutral rotation for 2 weeks; a gentle active motion was then initiated with a hinged brace for another 4 weeks. After that, the hinged brace was used only intermittently without range of motion limitations. Daily and occupational activities were allowed at 3 months after surgery.

### Functional and radiographic assessment

Postoperatively, all patients attended regular outpatient clinic follow-up appointments at 2 weeks, 3 months, 6 months, 1 year, and 2 years. The Mayo Elbow Performance Score (MEPS) was used for functional survey and the Shortened Disability of the Arm and Shoulder and Hand (quickDASH) score was used for the subjective disability evaluation. The MEPS is a widely-used, physician-based elbow performance index for evaluating clinical outcomes of a variety of elbow disorders and showed validated reliability and accuracy for evaluating the treatment results of elbow fractures and dislocation [8]. Being a shortened version of the DASH Outcome Measure, the quickDASH uses 11 items (scored 1–5) instead of 30 items as in the DASH measurement to evaluate perceived physical function and symptoms in people with musculoskeletal disorders of the upper limb [9]. A radiographic examination was performed next to surgery day, and 3 months, 1 year, and 2 years after surgery.

### Statistical analysis

Functional outcomes according to MEPS and quickDASH score were compared between different subtypes and between group A and B using an independent t-test. *P* values < 0.05 were considered statistically significant. Interobserver consistencies in radiographic diagnoses were analyzed between two observers using Cohen’s kappa coefficient (kappa) analysis, in which a kappa of 0.41–0.60 meant moderate agreement and a kappa > 0.60 indicated substantial agreement.

## Results

All patients underwent a clinical survey at an outpatient clinic with a mean follow-up of 2.3 years (range, 2–3.8 years). Radiographic union was defined by surgeon and radiologist consensus and was achieved in all cases at about 3 months after surgery. A functional survey was performed at 2 years with a mean MEPS of 87.75 ± 12.51 (range, 55–100) and quickDASH score of 7.05 ± 6.19 (range, 0–22). On MEPS grading, excellent results were achieved in 10 cases, while good results were achieved in eight. Fair and poor outcomes were achieved in one case each.

**Fig 5:**
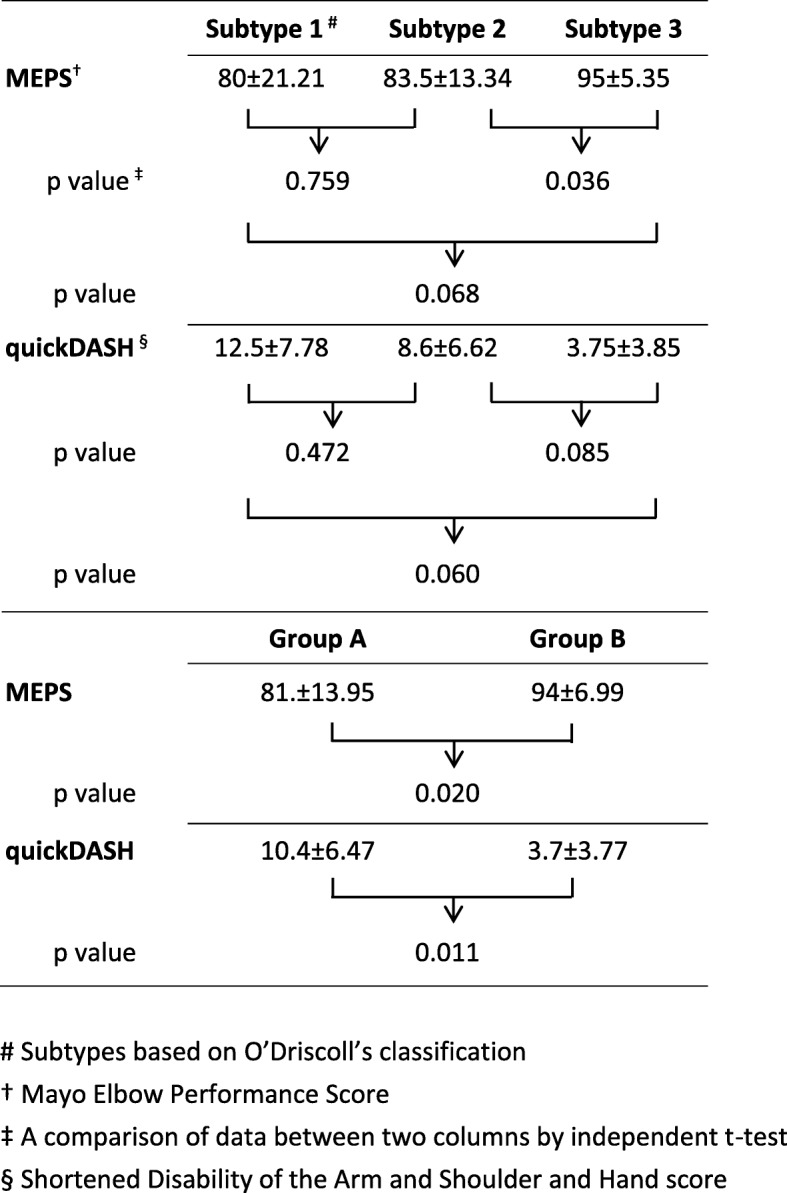
Comparison of functional assessment according to MEPS and DASH scores

Comparisons of different subtypes and radiographic groups are detailed in Fig. 5. Better functional outcomes were found in subtype 3 than in subtype 2, with a significant difference in MEPS (*p* = 0.036) but not in quickDASH scores (*p* = 0.85). The differences in functional scores between subtypes 1 and 2 and between subtypes 1 and 3 were insignificant. Functional outcomes were also compared between groups A and B. Significantly better outcomes were found in group B than in group A in both MEPS (*p* = 0.020) and quickDASH score (*p* = 0.011).

There were no immediate surgical complications such as neurovascular injury, wound problem, infection, or secondary displacement after fixation. Three cases of preoperative ulnar neuropathy were treated surgically with nerve transposition and achieved neurological recovery. No residual lateral instability was found after 3 months of follow-up. There were two cases of inferior outcomes: one received surgical fixation 1 week after acute trauma and had heterotopic ossification along the medical capsule postoperatively with a final range of motion of 5–80° and showed poor outcome on MEPS grading, while the other was a revision case that was treated with coronoid fixation at 3 weeks after the primary surgery. Partial resorption of the AMF fragment after revision surgery was found, and the final motion arc was 5–95° with residual instability.

Analysis of interobserver consistency on radiographic readings showed moderate agreement in the O’Driscoll classification (kappa = 0.56) and substantial agreement in the categorization of additional proximal ulnar fractures (kappa = 0.76). In cases of disagreement, reports of fracture classification and the categorization of additional fractures were empirically based on the surgeon’s recommendations.

## Discussion

The ulnar coronoid is a critical stabilizer in elbow trauma [10], and fractures involving > 25% of the coronoid height resulted in significant elbow instability [11]. O’Driscoll proposed a new classification of coronoid process fracture based on fracture size and location [5]. Reflecting the elbow injury mechanism, the O’Driscoll classification became popularly adopted as treatment guidance [12, 13]. Unlike the common fracture pattern of the anterior coronoid in terrible triad injuries, AMF fracture corresponding to O’Driscoll classification type 2 fracture shows a diverse presentation in fracture patterns and locations. Cases of type 2 fractures were further divided into three subtypes to cover different presentations of AMF fractures involving the coronoid tip, anteromedial cortex, sublime tubercle, or a combination thereof. Resulting from varus rotational injury force, AMF fractures could be associated with LCL insufficiency and lead to VPMRI [14].

The surgical approach to VPMRI includes fracture fixation, ligament repair, or both, but treatment priority has yet to be determined. A recent comparison study consisting of 18 patients with O’Driscoll type 2 fractures proposed a strategic approach to fix big coronoid fragment with or without ligament repair but to leave the small fragment alone [15]. We believed all the bony constructs should be repaired as possible, and adopted the “fracture-fixation” strategy that meant we fixed all the coronoid and proximal ulnar fractures first. Small or comminuted coronoid fragments were buttressed with low-profile plates and augmented by anchor or transosseous suture. After restoration of bony constructs, we then verified stability and repaired the LCL whenever residual instability persisted. Since AMF is mechanically considered the primary constraint against varus rotatory force in the elbow joint [6], anatomical restoration with secure fixation was recommended and critical in the treatment of AMF fractures with or without LCL insufficiency. Although a previous biomechanical study [16] and clinical report [17] documented that the direct management of LCL per se could improve the instability associated with AMF fractures, consensus is lacking regarding LCL injury severity. While surgical indications and LCL repair timing have yet to be established, LCL repair alone may not be beneficial whenever surgical reduction and secure fixation are achievable.

Despite the establishment of comprehensive classification systems and increased understanding of the pathophysiology, a definite treatment protocol and optimal surgical strategy have yet to be determined. In addition to the fracture patterns described in the O’Driscoll type 2 coronoid fracture, there could be various additional lesions over the proximal ulna. A recent study using CT images and a fracture mapping technique demonstrated considerable variability in coronoid fracture patterns and thus recommended that the present classification was insufficient for predicting coronoid fracture type [18]. Radiographs in half of our cases showed concomitant proximal ulnar fractures including olecranon, coronoid base, and coronoid articular depression fractures. All of those additional lesions were considered an integral part of VPMRI that needed to be fully addressed during surgical management. To our knowledge, the present study was the first to discuss the radiographic correlations of concomitant proximal ulnar injuries with AMF fractures and compared their treatment results. Significantly better functional outcome in cases of concomitant proximal ulnar lesions supported our surgical strategy and may encourage the surgeon to address all of the injured components in addition to AMF fractures. A more extended radiographic classification to include the proximal ulnar lesions is essentially important to categorize the injury patterns of AMF fractures. The use of a combined analysis of preoperative radiographic findings and injury mechanisms may enable surgeons to develop a meticulous surgical plan and thorough intervention of AMF fractures and the associated VPMRI.

This study had several limitations. First, it was a retrospective study from a consecutive series of 20 cases. Second, despite being a case-comparison study, the surgical approach and fixation modality was empirically determined through the planning and experience of a single surgeon. Third, there was a relatively small sample size in each group. Since we treated only referred cases from the primary trauma unit of our hospital and other institutes, a delay in definite treatment could have an adverse effect in either group. For future investigations, comparison studies with larger case numbers are necessary to further categorize associated injuries and clarify the critical role of the lateral elbow tissue in AMF fracture and VPMRI.

## Conclusions

An innovative surgical strategy was proposed according to radiographic findings. With consideration of additional proximal ulnar lesions, AMF fractures could be further classified and thoroughly treated before proceeding to the intraoperative varus stress test to determine the indication for LCL repair. Significantly better functional outcomes were found when the associated proximal ulnar lesions were fully addressed during the surgical management of AMF fractures.

## Abbreviations

AMF: Anteromedial facet
CT: Computed tomography
DASH: Shortened Disability of the Arm and Shoulder and Hand
LCL: Lateral collateral ligament
MEPS: Mayo Elbow Performance Score
VPMRI: Varus posteromedial rotatory instability
